# Using network pharmacology to explore the mechanism of Danggui-Shaoyao-San in the treatment of diabetic kidney disease

**DOI:** 10.3389/fphar.2022.832299

**Published:** 2022-08-19

**Authors:** Jinfei Yang, Chenrui Li, Yan Liu, Yachun Han, Hao Zhao, Shilu Luo, Chanyue Zhao, Na Jiang, Ming Yang, Lin Sun

**Affiliations:** Hunan Key Laboratory of Kidney Disease and Blood Purification, Department of Nephrology, The Second Xiangya Hospital of Central South University, Changsha, Hunan, China

**Keywords:** Danggui-Shaoyao-San, diabetic kidney disease, network pharmacology, inflammation, oxidative stress

## Abstract

Danggui-Shaoyao-San (DSS) is one of traditional Chinese medicine, which recently was found to play a protective role in diabetic kidney disease (DKD). However, the pharmacological mechanisms of DSS remain obscure. This study would explore the molecular mechanisms and bioactive ingredients of DSS in the treatment of DKD through network pharmacology. The potential target genes of DKD were obtained through OMIM database, the DigSee database and the DisGeNET database. DSS-related targets were acquired from the BATMAN-TCM database and the STITCH database. The common targets of DSS and DKD were selected for analysis in the STRING database, and the results were imported into Cytoscape to construct a protein-protein interaction network. Kyoto Encyclopedia of Genes and Genomes (KEGG) pathways enrichment analysis and Gene Ontology (GO) enrichment analysis were carried out to further explore the mechanisms of DSS in treating DKD. Molecular docking was conducted to identify the potential interactions between the compounds and the hub genes. Finally, 162 therapeutic targets of DKD and 550 target genes of DSS were obtained from our screening process. Among this, 28 common targets were considered potential therapeutic targets of DSS for treating DKD. Hub signaling pathways including HIF-1 signaling pathway, TNF signaling pathway, AMPK signaling pathway, mTOR signaling pathway, and PI3K-Akt signaling pathway may be involved in the treatment of DKD using DSS. Furthermore, TNF and PPARG, and poricoic acid C and stigmasterol were identified as hub genes and main active components in this network, respectively. In this study, DSS appears to treat DKD by multi-targets and multi-pathways such as inflammatory, oxidative stress, autophagy and fibrosis, which provided a novel perspective for further research of DSS for the treatment of DKD.

## Introduction

Diabetic kidney disease (DKD) is one of the most common microvascular complications of diabetes and is the leading cause of end-stage renal disease (Tuttle et al., 2014). Pathologically, DKD is characterized by glomerular basement membrane thickening, mesangial expansion, glomerulosclerosis and tubulointerstitial changes (Tervaert et al., 2010). A great number of studies have shown that the pathogenesis of DKD involves multiple mechanisms, including oxidative stress, inflammation, fibrosis and lipid accumulation (Liang et al., 2018; Tanaka et al., 2018; Chen X et al., 2019). Although existing treatments can slow down the progression of DKD in some patients, additional potent treatments need to be explored to fight against DKD.

Traditional Chinese medicine has been widely applied in the treatment of disease for thousands of years, and the application scope of many traditional Chinese medicine has been expanding (Tong et al., 2018; Peng et al., 2020). Danggui-Shaoyao-San (DSS) consists of *Paeonia lactiflora* Pall., *Alisma orientalis* (Sam.) Juzep., *Angelica sinensis* (Oliv.) Diels., *Poria cocos* (Schw.) Wolf., *Atractylodes macrocephala* Koidz. and *Ligusticum chuanxiong* Hort. In 2009, Chen et al. (2009) reported that they identified 41 components in DSS extract and determined 14 bioactive components. In addition, Yin et al. (2021) found that different solvents yield different extracts with different compounds and the extracts from DSS improved metabolic syndrome symptoms and the biochemical factors by regulating intestinal floras and improving hepatic gene expressions and metabolites. Actually, DSS used to be primarily used to treat gynecological diseases (Kotani et al., 1997; Lee et al., 2016). Subsequently, researchers have demonstrated that DSS can be applicable to ameliorate neurological disorders including Alzheimer’s disease (Huang et al., 2020; Kim and Cho, 2020; Li et al., 2021). Fu et al. (2022) recently reviewed that polysaccharide extracted from DSS play significant roles in the treatment of Alzheimer’s disease through various mechanisms, such as anti-inflammatory, anti-neuronal injury, and immune regulation. Furthermore, recent studies have showed that DSS may play an important role in the treatment of diabetes and its complications (Xiaobing et al., 2020; Shi et al., 2021). Especially, Liu et al. (2012) found that DSS has the renal protective effects on STZ-induced diabetic rats. And Yang et al. (2022) screened bioactive compounds from DSS for treating sodium retention in nephrotic syndrome and found that 1,2,3,4,6-O-pentagalloylglucose is a potential bioactive ingredient responsible for the effect of DSS on natriuresis. However, the bioactive components and molecular mechanisms of DSS as related to DKD remained unclear due to the limited research. Therefore, the aim of this study was to explore the potential key pharmacologic mechanisms of DSS on DKD.

Network pharmacology is an emerging discipline that integrate virtual computing technology, public databases, and high-throughput data for revealing the complicated network relationship between drugs and diseases. Nowadays, many researchers have used network pharmacology to study the therapeutic targets of traditional Chinese medicine, laying a foundation for the clinical application of traditional Chinese medicine (Qin et al., 2020; Zhou et al., 2021). DSS, as a traditional Chinese medicine, have multiple active compounds, however, its bioactive components and molecular mechanisms for the treatment of DKD needs to be further explored. In this study, the network pharmacology based on a compound-target-disease interaction network and biologic analysis were used to explore the potential mechanisms of DSS in the treatment of DKD and provide a basis for subsequent pharmacologic experimental research.

## Materials and methods

### Identifying diabetic kidney disease-Related Targets

Human genes associated with DKD were gathered from the OMIM database (http://omim.org) (Amberger et al., 2015), the DigSee database (http://210.107.182.61/geneSearch/) (Kim et al., 2013; Kim et al., 2017) and the DisGeNET database (https://www.disgenet.org/) (Piñero et al., 2015; Piñero et al., 2020). Terms like (diabetic and nephropathy) or (“diabetic nephropathy”) or (“diabetic kidney disease”) were used as baits to search DKD-related targets from the OMIM database. By removing duplicate items, 37 genes were obtained. The keywords “Diabetic nephropathy” or “Diabetic nephropathies” were used in the DisGeNET database or the DigSee database to screen targets related to DKD. Genes with ‘Score_gda’ > 0.25 in the DisGeNET database or number of evidence articles >5 in the DigSee database were selected for further analysis. Seventy-four DKD-related genes and 91 DKD-related genes were collected from these two databases, respectively. All genes were then inputted into UniProt (https://www.uniprot.org/) and converted into a unified gene name (UniProt, 2019). After deleting duplicates, 162 genes were identified as DKD targets finally.

### Active ingredients

DSS prescription consists of the following herbs: *Paeonia lactiflora* Pall., *Alisma orientalis* (Sam.) Juzep., *Angelica sinensis* (Oliv.) Diels., *Poria cocos* (Schw.) Wolf., *Atractylodes macrocephala* Koidz. and *Ligusticum chuanxiong* Hort. Active ingredients of every herb were mainly accessed through the TCMSP database (https://tcmspw.com/tcmsp.php), a platform provides information about Chinese herbal medicine, related targets and ingredients-targets-diseases networks (Ru et al., 2014). Names of herbs were inputted into the search box to obtain the ingredients of DSS. And the ingredients meeting following criterions were identified as pivotal active ingredients: 1) oral bioavailability ≥30%; 2) drug-likeness ≥ 0.18; 3) number of rotatable bonds ≤10; 4) molecular weight: 180-500.

### Predicting Danggui-Shaoyao-San-Related Targets

Targets of DSS active ingredients were acquired from the BATMAN-TCM database (http://bionet.ncpsb.org/batman-tcm/) (Liu et al., 2016) and the STITCH database (http://stitch.embl.de/) (Szklarczyk et al., 2016). Results were limited to “*Homo sapiens*.” Targets predicted from the BATMAN-TCM database were accepted for subsequent analysis under the retrieval of score >10. All obtained targets were standardized through the UniProt (https://www.uniprot.org/) (UniProt, 2019). Finally, 550 targets were predicted as DSS corresponding targets.

### Identifying ingredients-disease common targets

The screened DKD targets and DSS active ingredients related targets were imported into TBtools (version 1.0686) (Chen C et al., 2020) and the overlaid targets were identified as potential DKD targets that may be targeted by DSS.

### Enrichment analysis and network construction

Kyoto Encyclopedia of Genes and Genomes (KEGG) pathways enrichment analysis and Gene Ontology (GO) enrichment analysis were conducted by online function annotation tool DAVID (https://david.ncifcrf.gov/, version 6.8) (Huang da et al., 2009a; Huang da et al., 2009b) and visualized by a data visualization website (http://www.bioinformatics.com.cn/). Protein-protein interaction (PPI) networks for DKD targets, DSS targets and common targets were established by STRING database (https://string-db.org/, version 11.0) (Szklarczyk et al., 2019). PPI network of DKD targets, DSS-active ingredients-targets network, DSS-active ingredients-common targets network and DSS-active ingredients-common targets-enriched KEGG pathways network were further constructed by Cytoscape (version 3.7.2). Cytohubba and MCODE tools were used to screen hub genes.

### Molecular docking simulation

Crystal structures of screened hub macromolecule receptors were obtained from PDB database (http://www.pdb.org) (Berman et al., 2002) and were then imported into Pymol software (version 2.4.0) for removal of water molecules, co-crystallized ligands and ions. Subsequently, hydrogens were added and gasteiger charges were computed by AutoDockTools (version 1.5.6). The structures were saved as PDBQT format files after AD4 type was assigned. 3D-structures of corresponding ingredients were downloaded from pubchem database (https://pubchem.ncbi.nlm.nih.gov/) and were converted into Mol2 format files by Open Babel GUI software (version 2.3.1 http://openbabel.org) (O'Boyle et al., 2011). Then the small molecule ligand files were imported into AutoDock Tools (version 1.5.6) and torsions were set automatically. The structures were then saved as PDBQT format files, too. Subsequently, PDBQT files of macromolecule receptors and corresponding small molecule ligands were imported into AutoDockTools (version 1.5.6) for construction of mating pockets. Further molecular docking simulation was conducted in AutoDockTools (version 1.5.6) by using Genetic algorithm. Conformations with the lowest docking binding free energy were selected as the most possible docking modes and visualized using PyMOL (version 2.4.0).

## 3 Results

### Diabetic kidney disease therapeutic targets, enriched pathways and PPI network

**FIGURE 1:**
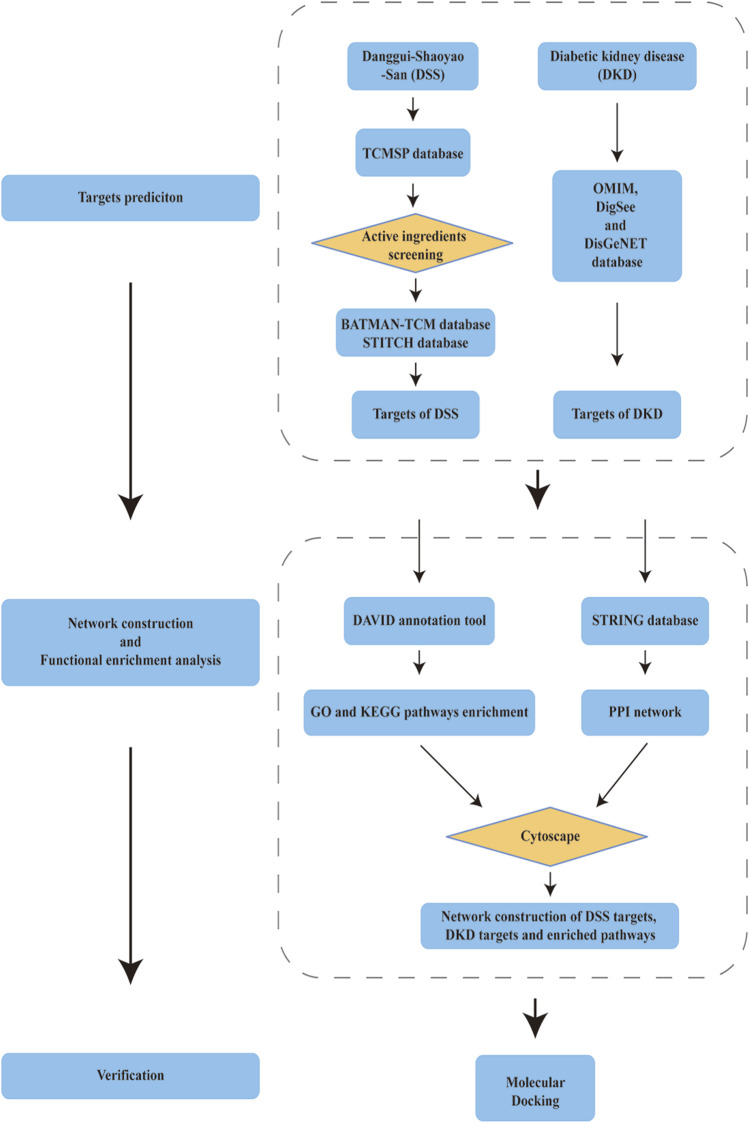
Schema of network pharmacology analysis of Danggui-Shaoyao-San (DSS) against diabetic kidney disease (DKD).

Flow chart of the network pharmacology is given in Figure 1. Briefly, DKD therapeutic targets and DSS targets were obtained from online databases, and the shared targets of these two parts were then identified as latent therapeutic targets of DSS against DKD. Function analysis and PPI network construction were then implemented to elucidate the potential mechanism of DSS in the treatment of DKD. Subsequent molecular docking was performed to further clarify the drug-target interactions.

**FIGURE 2:**
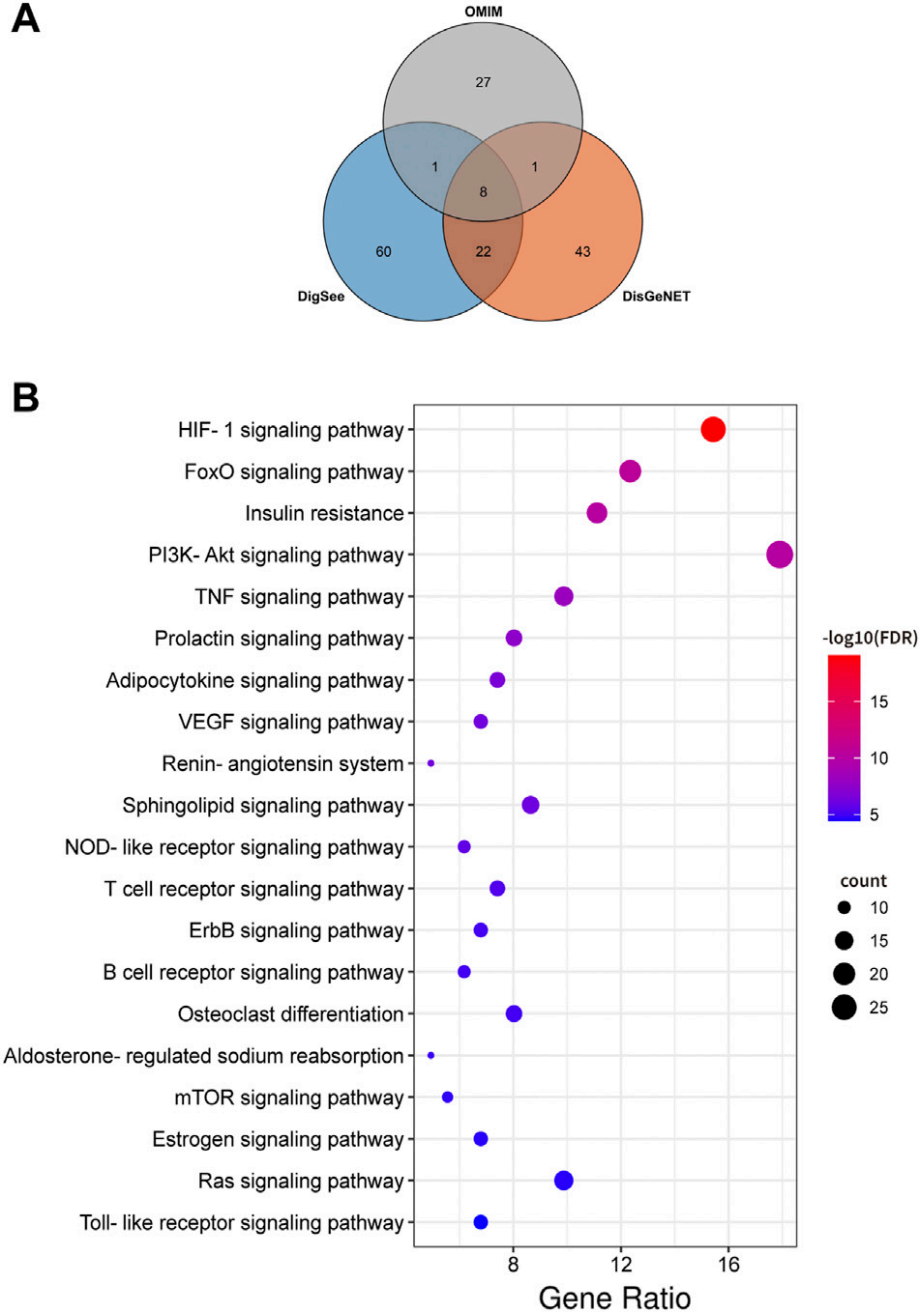
DKD targets and Kyoto Encyclopedia of Genes and Genomes (KEGG) enrichment analysis. **(A)** Venn diagram composed of the OMIM database retrieved DKD targets, DigSee database retrieved DKD targets and DisGeNET database retrieved DKD targets. **(B)** Dot bubble plot of the top 20 KEGG pathways enriched by the targets of DKD. The size of these dots represents the number of enriched genes, and the color represents the −log_10_ (FDR).

**FIGURE 3:**
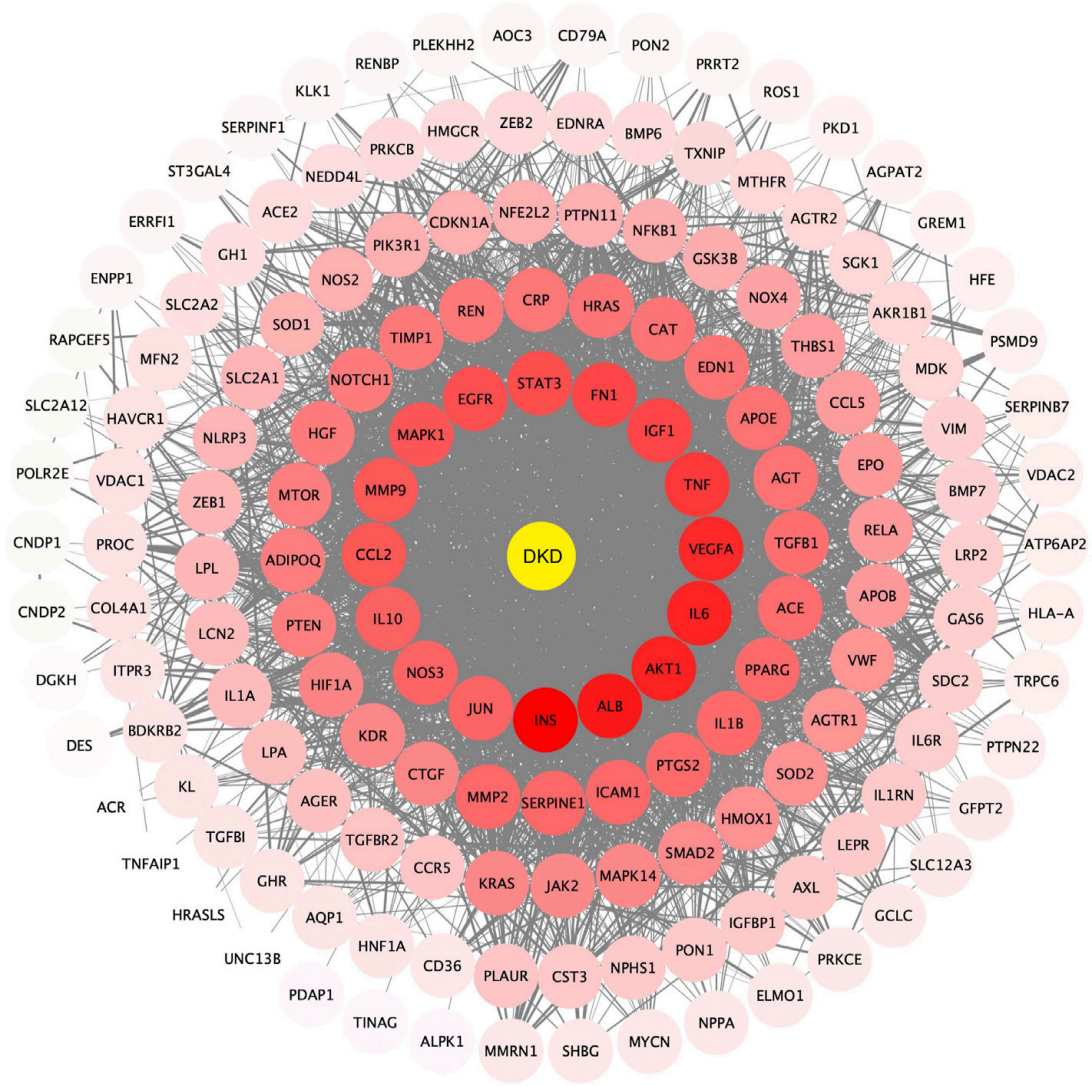
Protein-protein interactions network of DKD targets. Color and location of one protein represent the number of connections with other proteins. The redder the color and the more central the position, the more interaction with other proteins. FDR: false discovery rate. DKD: diabetic kidney disease.

An aggregate of 162 targets were screened as DKD therapeutic targets from OMIM, DigSee and DigSee database (Figure 2A). KEGG enrichment analysis of these genes showed that pathways, including HIF-1 signaling pathway, PI3K-AKT signaling pathway, VEGF signaling pathway, TNF signaling pathway and NOD-like receptor signaling pathway, participated in the development of DKD (Figure 2B). A PPI network of these targets were then constructed. One hundred and sixty DKD potential therapeutic targets nodes and 2,465 protein-protein edges were present in Figure 3. According to the number of interactions between points, we sorted these disease targets. The points closing to the center and being redder indicate more numbers of interactions between points and other points and its importance. As presented in Figure 3, targets like INS, ALB, AKT1, IL6, VEGFA and TNF might be key targets of DKD.

### Danggui-Shaoyao-San targets, function, and protein-protein interaction network

**FIGURE 4:**
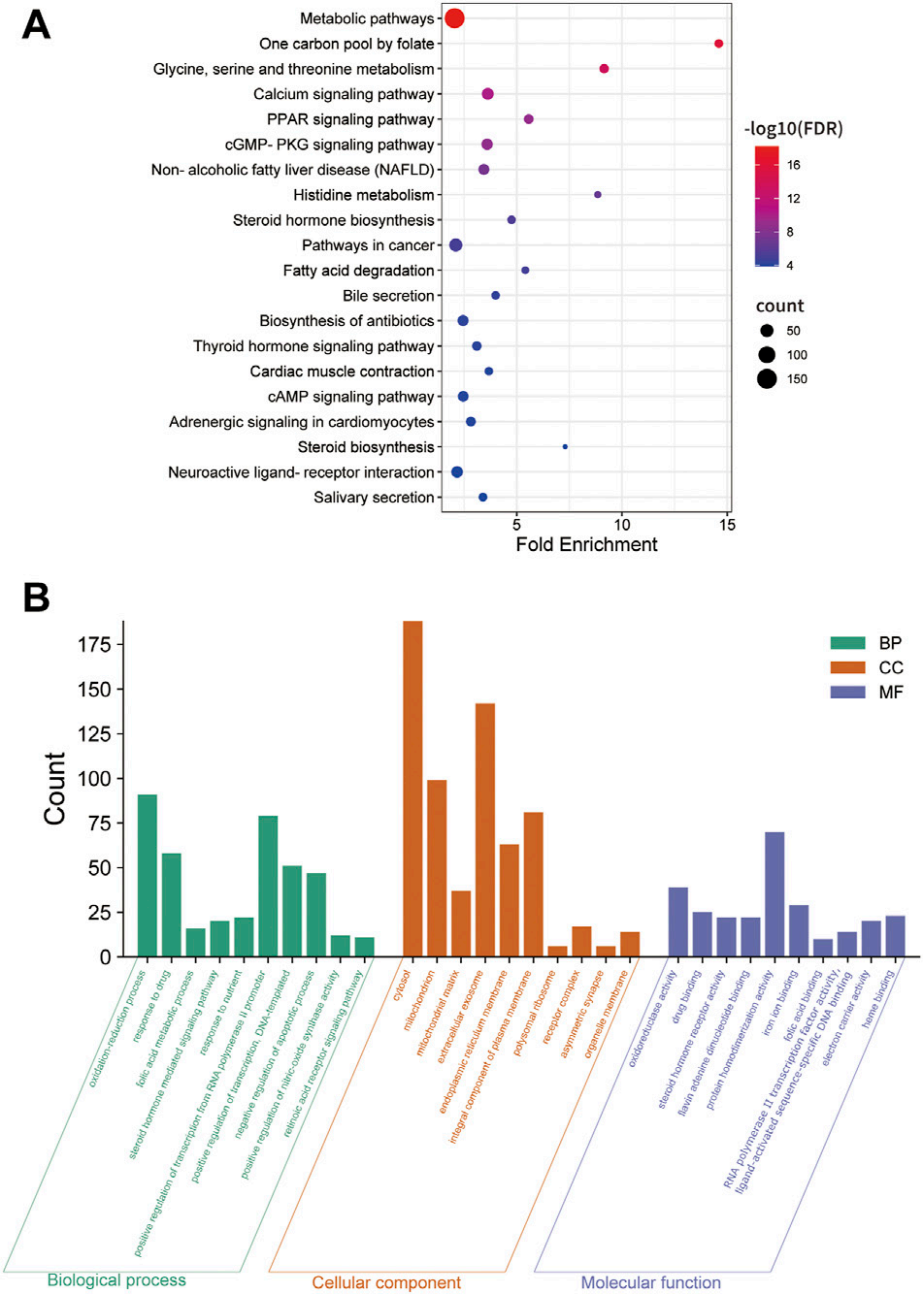
KEGG enrichment, Gene Ontology (GO) enrichment of the DSS targets. **(A)** Dot bubble plot of the top 20 KEGG pathways enriched by the predicted targets of DSS. The size of these dots represents the number of enriched genes, and the color represents -log_10_ (FDR). **(B)** Histogram plot of the top 10 enriched molecular function, cellular component and biological process of DSS targets. BP: biological processes; CC: cellular components; MF: molecular functions.

**FIGURE 5:**
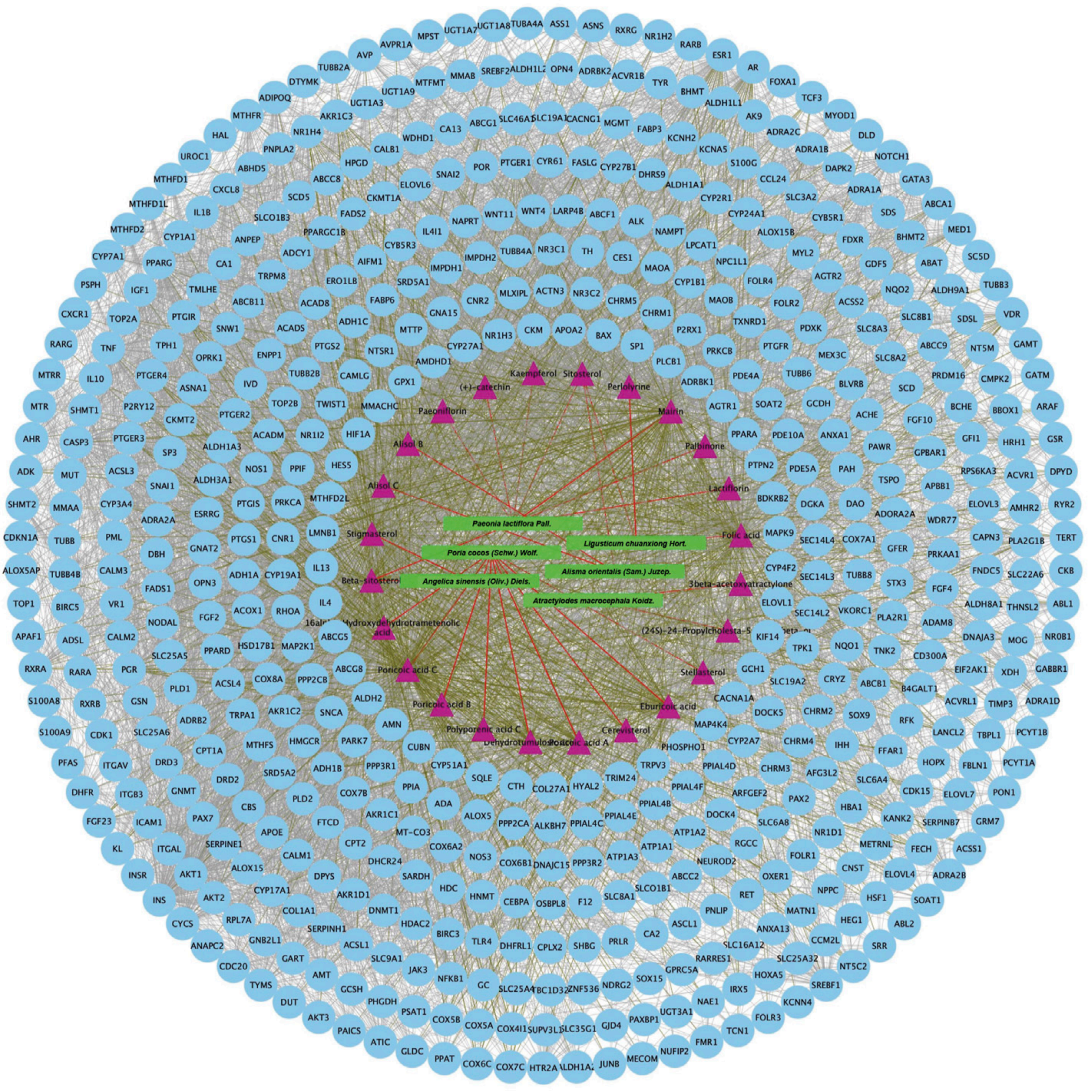
Protein-protein interaction network of proteins targeted by 24 active chemical ingredients of DSS. The green rectangles represent herbs of DSS, the purple triangles represent screened active ingredients of these herbs, and the blue circles represent their targets. The red lines represent the interactions between herbs and the chemical ingredients, the grass green lines represent the interactions between ingredients and the predicted targets, while the protein-protein interactions were represented as prey lines.

Twenty-four active components of 6 Chinese herbal medicines were recognized according to the screening conditions. And 550 targets of these active components were further predicted by the BATMAN-TCM database and the STITCH database. KEGG enrichment analysis suggested that these targets of DSS are involved in a variety of metabolic pathways, such as glycine, serine and threonine metabolism, histidine metabolism, and fatty acid degradation (Figure 4A). Besides, PPAR signaling pathway, cGMP-PKG signaling pathway, and cAMP signaling pathway were also been identified (Figure 4A). Other important terms like inflammatory mediator regulation of TRP channels, insulin resistance, apoptosis, HIF-1 signaling pathway, Rap1 signaling pathway, AMPK signaling pathway, and mTOR signaling pathway are also enriched by these targets (data not show). GO enrichment analysis for molecular function indicated that targets of DSS possess protein homodimerization activity, heterodimerization activity and oxidoreductase activity (Figure 4B). Furthermore, these targets were also enriched in enzyme binding, transcription factor binding and other molecular functions (Figure 4B). GO enrichment analysis for cellular component implied that most of these targets were located in cytosol and mitochondrion (Figure 4B). GO enrichment analysis for biological process showed that these active components of DSS were possibly associated with oxidation-reduction process, response to drug, apoptotic process and so on (Figure 4B). Moreover, we constructed a network composed of traditional Chinese herbal medicines of DSS, active ingredients, targets and interactions between targets (Figure 5).

### Danggui-Shaoyao-San-diabetic kidney disease common targets

**FIGURE 6:**
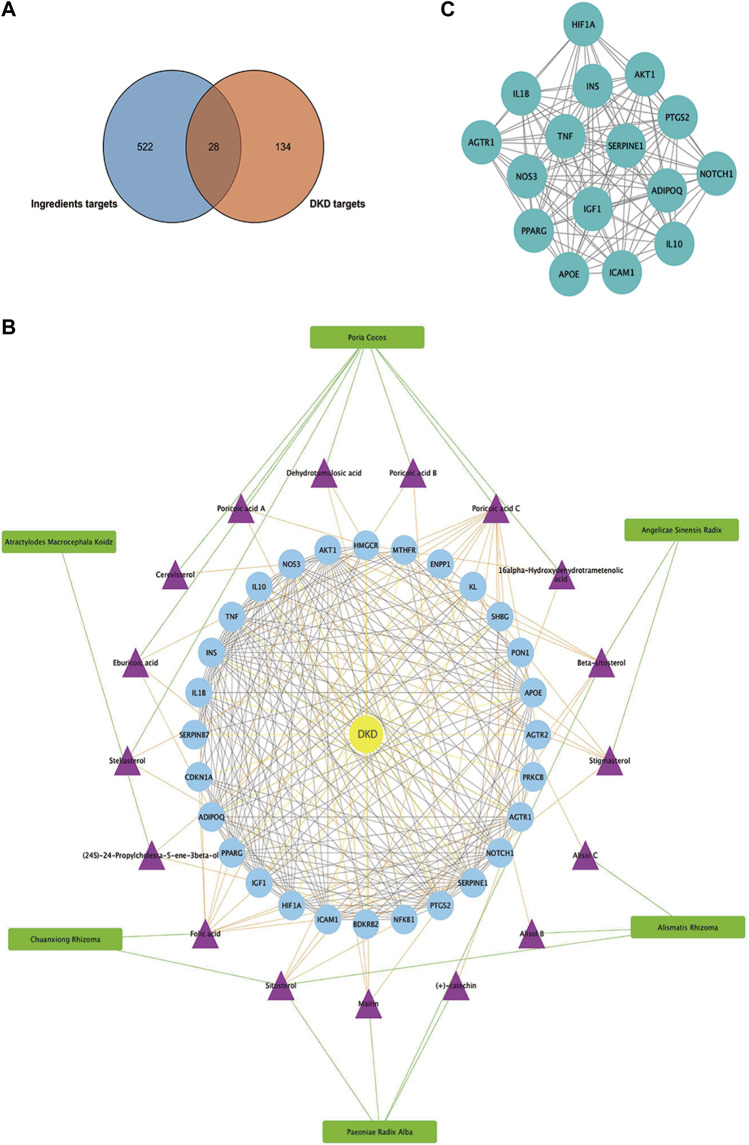
Matching of target genes between DKD and DSS, and network analysis of the common targets. **(A)** Venn diagram composed of the ingredients targets and DKD targets. **(B)** Herbs-active ingredients-common targets-Disease network. The green rectangles represent herbs of DSS, the purple triangles represent screened active ingredients of these herbs, and the blue circles represent the common targets of DSS and DKD. The green lines represent the interactions between herbs and the chemical ingredients, the orange lines represent the interactions between ingredients and the common targets, the yellow lines represent interactions between DKD and the common targets, while the protein-protein interactions were represented as prey lines. **(C)** Protein-protein interaction network constructed of 16 core targets screened by MCODE tools.

**FIGURE 7:**
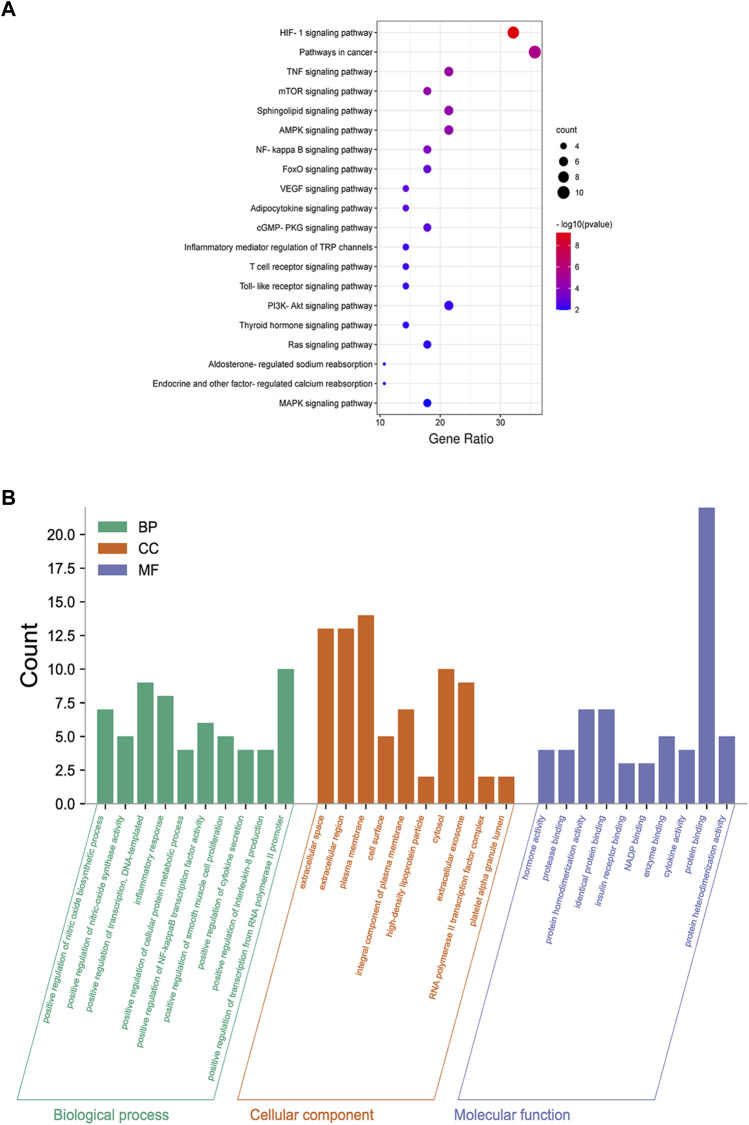
Functional enrichment analysis of common targets. **(A)** Dot bubble plot of the top 20 KEGG pathways enriched by the common targets of DKD and DSS. Size of the dots represents the number of enriched genes, and the color represents −log_10_ (*p* value). **(B)** Histogram plot of the top 10 biological processes, cellular components and molecular functions of common targets by GO enrichment analysis. BP: biological processes; CC: cellular components; MF: molecular functions.

**FIGURE 8:**
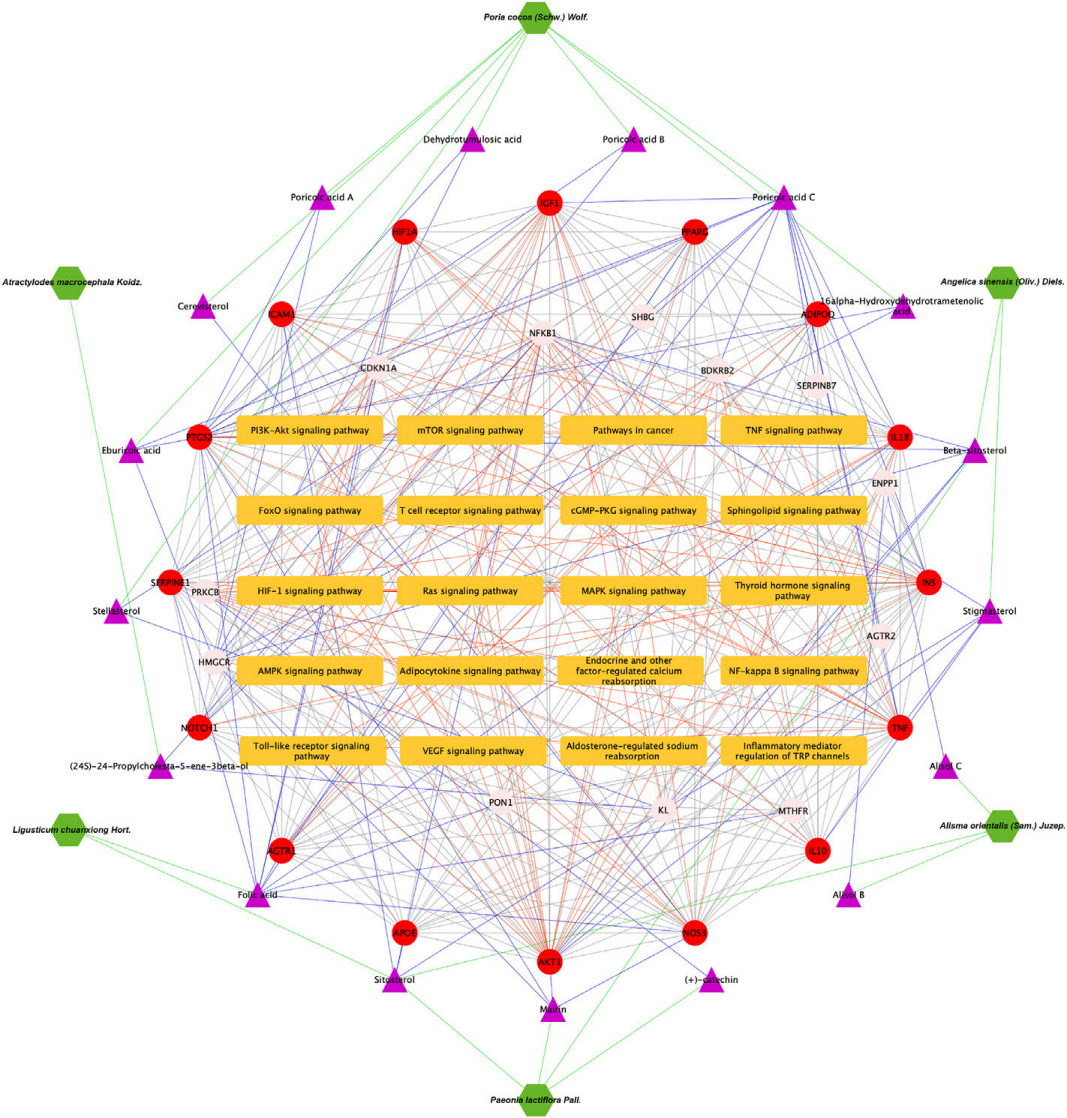
Herbs-active ingredients-common targets-enriched KEGG pathways network. Green hexagons represent herbs of DSS, the purple triangles represent screened active ingredients of these herbs, the red circles represent the 16 core common targets of DSS and DKD, pink circles represent other common targets, and the orange rectangles represent enriched KEGG pathways. The green lines represent the interactions between herbs and the chemical ingredients, the blue lines represent the interactions between ingredients and the common targets, the red lines represent interactions between the common targets and KEGG pathways, while the protein-protein interactions were represented as prey lines.

As presented in Figure 6A, 28common targets were identified as DKD related proteins targeted by DSS. Subsequently, we constructed a network consisting of DSS herbs, active ingredients, common targets, interactions between targets and disease (Figure 6B). Based on the PPI network of common targets, 16 core target genes including HIF1A, TNF, IL1B, IL10, ICAM1, NOTCH1, INS, AKT1, AGTR1, SERPINE1, PTGS2, NOS3, IGF1, ADIPOQ, PPARG, and APOE were recognized by Cytoscape MCODE tool (Figure 6C; Table 1). In order to further clarify the function of these common genes, KEGG pathway enrichment analysis was carried out. As shown in Figure 7A, DSS may ameliorate renal damage of DKD through HIF-1 signaling pathway, TNF signaling pathway, mTOR signaling pathway and so on. GO enrichment analysis indicated that these common targets may be associated with nitric oxide biosynthetic, inflammatory response, NF-kappa B transcription factor activity and other important biological processes (Figure 7B). Besides, these common targets located in extracellular space, plasma membrane and cytosol most (Figure 7B). And GO enrichment analysis for molecular function showed that these targets may be related to hormone activity, protease binding, insulin receptor binding and so on (Figure 7B). To better understand the relationship between these drug-disease common targets and KEGG pathways, we constructed a network composed of herbs, chemical ingredients, common targets and KEGG pathways Figure 8. And the relationships among these chemical ingredients, targets and KEGG pathways were also summarized in Supplemenatry Table S1.

**TABLE 1:** Screened hub genes by Cytohubba and MCODE and corresponding active ingredients.

Target gene	MCC score by cytohubba	Top protein-protein interaction network in MCODE analysis	MCODE score	Corresponding active ingredients
TNF	7186125008	Y	13	Stigmasterol, Poricoic acid C
PPARG	7186123440	Y	13	Poricoic acid C, Eburicoic acid, Folic acid
AKT1	7186118406	Y	13	Mairin, Stigmasterol, Beta-sitosterol
IL1B	7186112646	Y	13	Poricoic acid C, Folic acid
PTGS2	7186112640	Y	13	Mairin, Beta-sitosterol, 16alpha-Hydroxydehydrotrametenolic acid, Poricoic acid C, Poricoic acid B, Dehydrotumulosic acid, Poricoic acid A, Eburicoic acid, Folic acid
INS	7185762136	Y	13	Poricoic acid C
IGF1	7185750600	Y	13	Poricoic acid C
ICAM1	7185386880	Y	13	Sitosterol
APOE	7185036240	Y	13	Sitosterol
NOS3	7185035534	Y	13	Mairin, Cerevisterol, Folic acid
HIF1A	6707111040	Y	13	Folic acid
SERPINE1	6706391880	Y	13	Folic acid
IL10	6706385286	Y	13	Stigmasterol
ADIPOQ	6706032600	Y	13	Poricoic acid C
NOTCH1	479727360	Y	12	Poricoic acid C
AGTR1	479002328	Y	12	Poricoic acid C, Folic acid
CDKN1A	725760	N	**—**	Folic acid
NFKB1	362880	N	**—**	Stigmasterol, Beta-sitosterol, Stellasterol, (24S)-24-Propylcholesta-5-ene-3beta-ol, Sitosterol
PON1	5762	N	**—**	Folic acid
HMGCR	5042	N	**—**	Mairin, Beta-sitosterol, 16alpha-Hydroxydehydrotrametenolic acid, Poricoic acid B, Dehydrotumulosic acid, Poricoic acid A, Eburicoic acid
MTHFR	1440	N	**—**	Folic acid
KL	722	N	**—**	Stigmasterol, Beta-sitosterol, Stellasterol, (24S)-24-Propylcholesta-5-ene-3beta-ol, Sitosterol
SHBG	120	N	**—**	Poricoic acid C
SERPINB7	8	N	**—**	Poricoic acid C
PRKCB	6	N	**—**	(+)-catechin
AGTR2	6	N	**—**	Poricoic acid C
ENPP1	4	N	**—**	Alisol B, Alisol C
BDKRB2	2	N	**—**	Poricoic acid C

Abbreviations: Y: yes; N: no.

### Pivotal intersection targets and molecular docking

**FIGURE 9:**
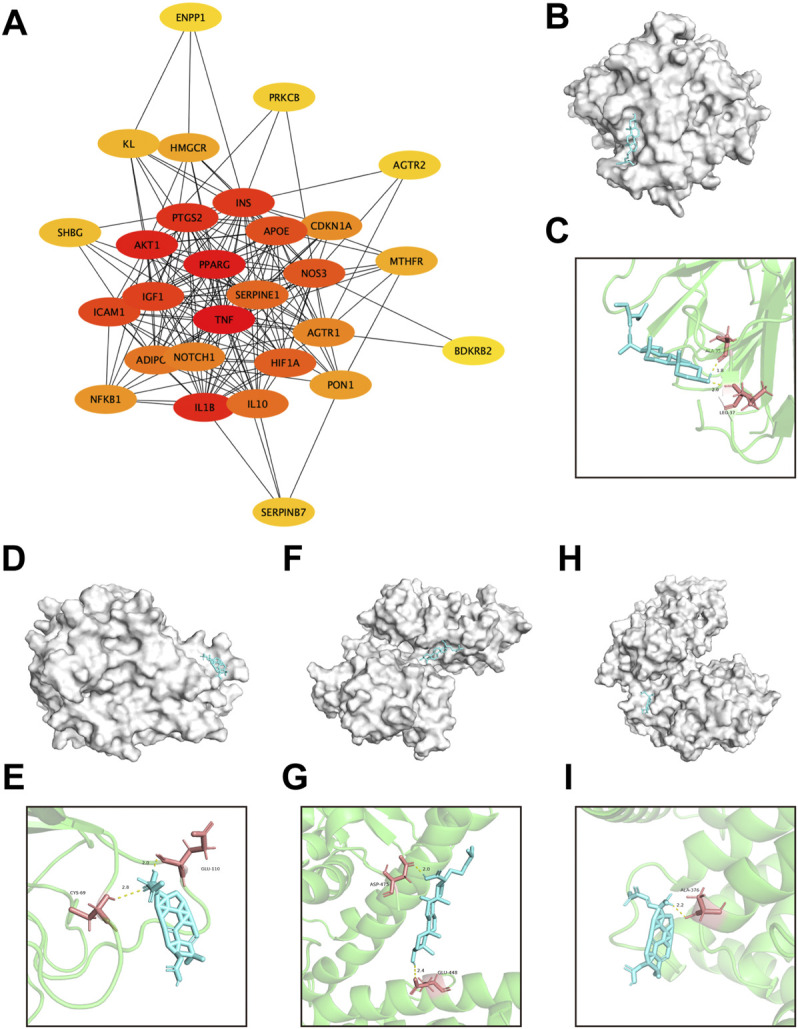
Screening of pivotal common targets and molecular docking of these targets binding to DSS ingredients. **(A)** Protein-protein interaction network of 28 common targets, ranked by Cutohubba tool. The redder the color, the more likely it is to be pivotal targets. **(B,C)** Molecular docking of stigmasterol with TNF. **(D,E)** Molecular docking of poricoic acid C with TNF. **(F,G)** Molecular docking of eburicoic acid with PPARG. **(H,I)** Molecular docking of poricoic acid C with PPARG. The blue small molecular structures represent the ingredients. And the white macromolecules represent screened important macromolecular receptors in panel **(B,D,F,H)**. The green structures in panel **(C,E,G,I)** represent screened macromolecular receptor structures, while the red sticks represent the interacted residuals, the yellow dotted line represent intermolecular hydrogen bonding.

Subsequently, the pivotal intersection targets were screened by Cytohubba tool in Cytoscape software. As showed in Figure 9A and Table 1, TNF, PPARG, ATK1 and IL1B were identified as hub genes in this network, and may play a critical role in the therapeutic effect of DSS against DKD, especially TNF and PPARG. Molecular docking of these 2 proteins and their predicted interacting DSS ingredients were performed then. The binding energy and binding activity of these interacting pairs were summarized in Table 2. The interaction between poricoic acid C and TNF requires the lowest docking binding free energy (−6.29 Kcal/Mol), followed by poricoic acid C and PPARG (−5.53 Kcal/Mol), and stigmasterol and TNF (−5.46 Kcal/Mol), indicating a good binding activity in the molecular docking, while the interaction between folic acid and PPARG was not certain (-2.6 Kcal/Mol). The interaction mode of stigmasterol and TNF was displayed in Figures 9B, C. As shown in Figure 9B, stigmasterol was positioned in the small groove on the surface of TNF. And Figure 9C suggested that stigmasterol may interact with TNF through ALA-35 residue and LEU-37 residue. Stigmastrol may be pulled to TNF through 2 hydrogen bonds and thus exerts the therapeutic potential (Figure 9C). Similarly, Figures 9D–I showed the molecular docking results of poricoic acid C with TNF, eburicoic acid with PPARG, and poricoic acid C with PPARG.

**TABLE 2:** DSS molecular docking binding enery results.

Receptors	Ligands	Binding energy (Kcal/Mol)	Binding activity
TNF	Stigmasterol	−5.46	Good
TNF	Poricoic acid C	−6.29	Good
PPARG	Eburicoic acid	−4.78	Certain
PPARG	Folic acid	−2.6	**—**
PPARG	Poricoic acid C	−5.53	Good

Note: Binding Energy less than −4.25 kcal/mol, −5.0 kcal/mol and −7.0 kcal/mol indicates a certain, good and strong binding activity, respectively.

## Discussion

Although there are many ways to slow the progression of DKD, there is still a lack of an effective way to prevent or cure it due to its complicate pathogenic mechanism. Therefore, it is urgent to find new and multi-effective drugs for the treatment of DKD from a broader perspective. DSS is one of promising drugs for DKD. Recent studies demonstrated that DSS plays a crucially reno-protective role in STZ-induced diabetic rats (Liu et al., 2012; Xiaobing et al., 2020). Unfortunately, the underlying mechanisms of DSS against DKD is still unknown. In this study, we investigate the potential molecular mechanism and active ingredients of DSS in the treatment of DKD based on network pharmacology.

In the current study, 28 DSS-DKD-common-targets were obtained and 16 core targets were acquired based on PPI network of these common targets by MCODE. The core targets include HIF1A, TNF, IL1B, IL10, ICAM1, NOTCH1, INS, AKT1, AGTR1, SERPINE1, PTGS2, NOS3, IGF1, ADIPOQ, PPARG, and APOE. Besides, KEGG pathways enrichment analysis of these 28 common targets found that DSS might ameliorate diabetic kidney injury through HIF-1 signaling pathway, TNF signaling pathway, mTOR signaling pathway and so on. Subsequent analysis by Cytohubba further ranked the DSS-DKD-common-targets. TNF and PPARG were identified as the most core targets.

TNF is a pro-inflammatory cytokine that promotes the expression of a variety of transcription factors, cytokines, cell adhesion molecules, mediators of inflammatory processes, and acute-phase proteins by binding to its receptors (Ortiz et al., 1995; Navarro and Mora-Fernández, 2006). TNF was also reported to be involved in the development and progression of DKD (Navarro-González and Mora-Fernández, 2008; Park et al., 2019). And TNF is able to induce diabetic renal injury by inducing apoptosis, the production of reactive oxygen species and so on (Laster et al., 1988; Radeke et al., 1990; Boyle et al., 2003). Other inflammatory factors like IL1B and IL10, as well as acute-phase protein marker of inflammation, ICAM1, were also found to be critical core targets of DSS against DKD. These targets have been proved to play an important role in the occurrence and development of DKD (Sangoi et al., 2016; Tang and Yiu, 2020). These proteins also participant in inflammation-related signaling pathways such as TNF signaling pathway and T cell receptor signaling pathway. Yin et al. (2021) also reported that DSS could reduce the expression of TNF in fructose-fed rats and Bi et al. (2021) demonstrated that DSS alleviates atherosclerosis through decreasing the expression of molecule including TNF, IL-1B and ICAM1. Hence, in this study, we found that DSS may improve DKD through inflammation-related signaling pathways by targeting TNF and other related targets, which was consistent with previous research.

PPARG is another important DKD-related gene. Previous study identified PPARG as a transcription factor modulating DKD target genes (Chen L et al., 2020). And Sharaf et al. (2018) found that PPARG showed lower expression in DKD patients and the abundance of PPARG were negatively correlated with microalbumin. Nevertheless, few studies have explored the specific role of PPARG in DKD. PPARG, a type II nuclear receptor, participates in fatty acid storage and glucose metabolism (Kökény et al., 2021). Recently, more and more researchers pay attention to the important role of abnormal lipid metabolism and renal ectopic fat deposition in the pathogenesis of DKD (Chen X et al., 2019; Yoshioka et al., 2021). In this study, we reported that DSS regulates a variety of metabolic pathways including PPAR signaling pathway. Consistently, Liu et al. (2022) also demonstrated that DSS regulates lipid metabolism though PPARG in scopolamine-induced amnesia. These findings indicated that DSS may play a reno-protective role in DKD through metabolism pathways by targeting PPARG, which is worthy to be validated by experiments in the future.

Further molecular docking of TNF and PPARG with their predicted active ingredients of DSS was conducted. Among them, the docking of poricoic acid C and TNF had the lowest binding energy (−6.29 Kcal/Mol), followed by poricoic acid C and PPARG (−5.53 Kcal/Mol), and stigmasterol and TNF (−5.46 Kcal/Mol), mirroring a good binding activity in the molecular docking. Poricoic acid C is a kind of triterpenoid compounds. Previous study found that poricoic acid A could protect against acute kidney injury-to-chronic kidney disease transition in rats and attenuate fibroblast activation and abnormal extracellular matrix remodeling in renal fibrosis (Chen DQ et al., 2019; Chen DQ et al., 2020). Poricoic acid B could decrease the production of cytokines (TNF, IL-1B, and IL-6) (Zhang et al., 2021). And our data showed that poricoic acid C might interact with TNF and thus ameliorate renal injury of DKD. On the other hand, Chen et al. (2009) demonstrated that poricoic acid could significantly decrease free fatty acid-induced intracellular triglyceride accumulation (Kim et al., 2019). And consistently, we predicted that poricoic acid C could interact with PPARG. Meanwhile, Stigmasterol is a natural with anti-inflammatory activity. Khan et al. (2020) found that stigmasterol significantly suppresses the inflammation by decreasing the expression of proinflammatory mediators (TNF, IL-6, IL-1B) and increasing the expression of anti-inflammatory cytokine (IL-10) in collagen-induced arthritis. And Kishore demonstrated that stigmasterol might inhibit the progression of DKD by ameliorating oxidative stress (Kishore et al., 2016). In general, our analysis shows that poricoic acid C and stigmasterol may play a protective role in DKD through anti-inflammatory and anti-lipid deposition *via* TNF and PPARG. However, there is no research on the role of poricoic acid C in DKD at present. Subsequent experiments can explore the reno-protective function of these active ingredients.

We also explore the potential mechanisms of DSS against DKD through functional enrichment analysis. KEGG pathways enrichment analysis suggests that DSS participates in the regulation of several important pathways, such as HIF-1 signaling pathway, TNF signaling pathway, mTOR signaling pathway, AMPK signaling pathway, NF-kappa B signaling pathway and so on, some of which were also key pathways of DKD. These pathways are closely related to hypoxia injury, inflammation and autophagy. Previous studies also reported that DSS alleviated kidney injury through its anti-oxidant effect (Liu et al., 2012). In cerebral ischemic-reperfusion injury, Luo et al. (2021) also found that DSS could attenuate oxidative stress and inhibit neuronal apoptosis. Besides, in lipopolysaccharide-induced human umbilical vein endothelial cells injury model, Bi et al. (2021) suggested that DSS could inhibit the phosphorylation of NF-kappa B. In addition, Zhang and others demonstrated that DSS improves renal fibrosis through attenuating hypoxia and regulating autophagy in unilateral ureteral obstruction rat model (Zhang et al., 2019). The above results are consistent with the pathways of DSS against DKD, strongly indicating that DSS is a promising drug for DKD treatment through regulating multiple cellular biological processes, such as inflammatory, oxidative stress, autophagy and fibrosis.

## Conclusion

**FIGURE 10:**
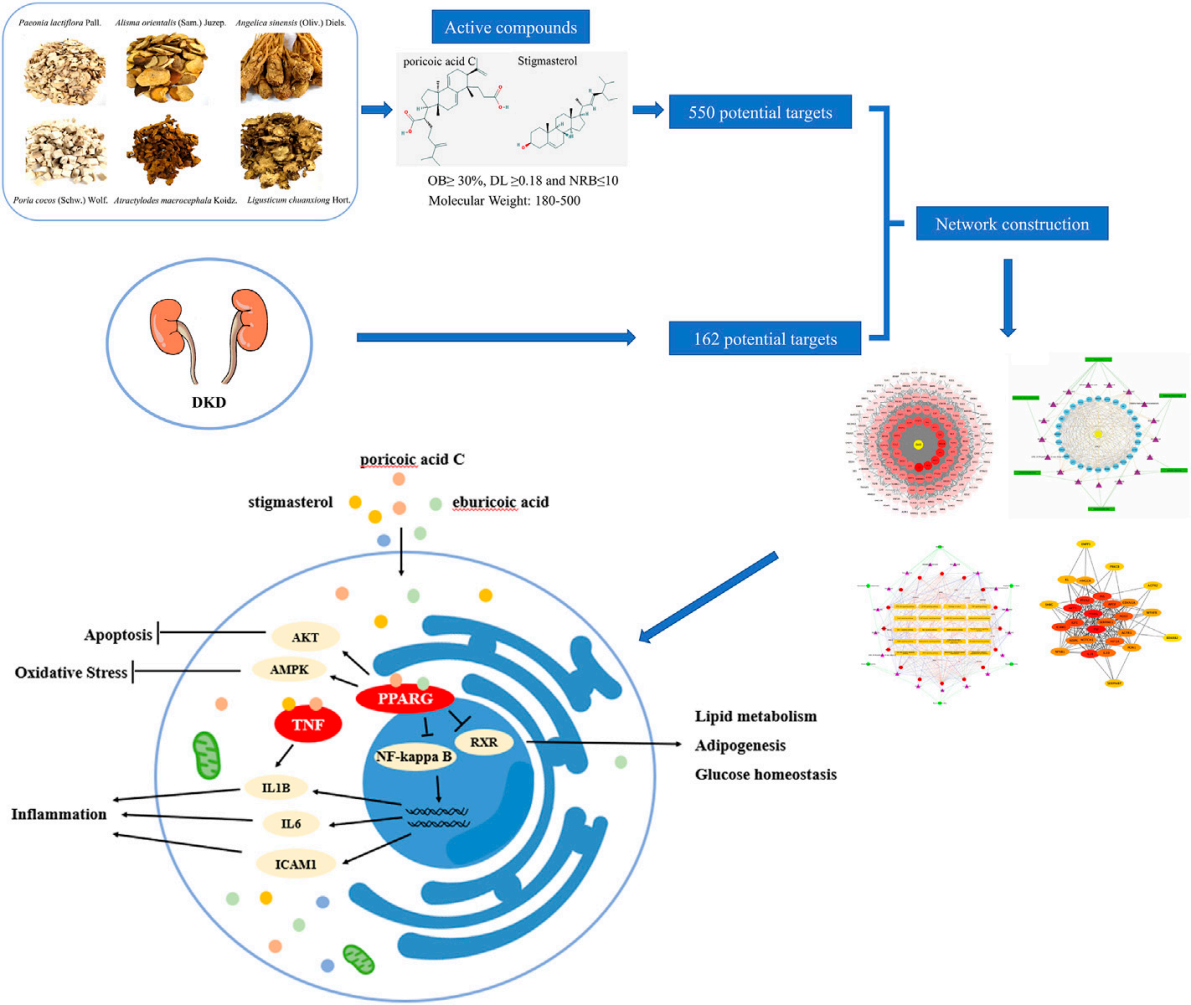
Schematic diagram of the network pharmacology for the pharmacological mechanisms of DSS for treatment of DKD.

The present study provides new insight into the pharmacology of DSS and the therapeutic mechanism for DKD, which also indicates that network pharmacology could be an effective way to explore the potential mechanism of intricate prescription for diseases. Our results using network pharmacology revealed that DSS play critically protective role in the treatment of DKD through multi-targets and multi-pathways (Figure 10).

## Data Availability

The original contributions presented in the study are included in the article/Supplementary Material, further inquiries can be directed to the corresponding author.

## Glossary

ADIPOQ: adiponectin
AGTR1: angiotensin II receptor type 1
AKT: protein kinase B
AKT1: AKT serine/threonine kinase 1
ALB: albumin
AMPK: adenosine monophosphate-activated protein kinase
APOE: apolipoprotein E
cAMP: cyclic adenosine monophosphate
cGMP: cyclic guanosine monophosphate
DKD: diabetic kidney disease
DSS: Danggui-Shaoyao-San
GO: Gene Ontology
HIF-1: hypoxia-inducible factor 1
HIF1A: hypoxia inducible factor 1 subunit alpha
ICAM1: intercellular adhesion molecule 1
IGF1: insulin like growth factor 1
IL10: interleukin-10
IL1B: interleukin-1 beta
IL6: interleukin-6
INS: insulin
KEGG: Kyoto Encyclopedia of Genes and Genomes
mTOR: mechanistic target of rapamycin kinase
NF-kappa B: nuclear factor-kappa B
NOD-like receptor: nucleotide-binding oligomerization domain-like receptors
NOS3: nitric oxide synthase 3
NOTCH1: notch receptor 1
PI3K: phosphatidylinositol 3-kinase
PKG: protein kinase G
PPAR: peroxisome proliferator-activated receptor
PPARG: peroxisome proliferator-activated receptor gamma
PPI: Protein-protein interaction
PTGS2: prostaglandin-endoperoxide synthase 2
Rap1: Ras-related protein 1
SERPINE1: serpin family E member 1
TNF: tumor necrosis factor
VEGF: vascular endothelial growth factor
VEGFA: vascular endothelial growth factor A
